# Adrenocorticotropic hormone-producing pituitary adenoma with pituitary apoplexy treated by surgical decompression: a case report

**DOI:** 10.1186/s12917-022-03502-2

**Published:** 2022-11-12

**Authors:** Sachiyo Tanaka, Shuji Suzuki, Mana Oishi, Satoshi Soeta, Ryosuke Namiki, Yasushi Hara

**Affiliations:** 1 Laboratory of Veterinary Surgery, Faculty of Veterinary Science, Nippon Veterinary and Life Science University, 1-7-1 Kyonancho, Musashino-shi, Tokyo, 180-8602 Japan; 2 Veterinary Medical Teaching Hospital, Nippon Veterinary and Life Science University, 1-7-1 Kyonancho, Musashino-shi, Tokyo, 180-8602 Japan; 3 Laboratory of Veterinary Anatomy, Faculty of Veterinary Science, Nippon Veterinary and Life Science University, 1-7-1 Kyonan, Musashino, Tokyo, 180-8602 Japan; 4 Gotokuji Namiki Animal Clinic, 1-50-15 Gotokuji, Setagaya, Tokyo, 154-0021 Japan

**Keywords:** Pituitary-dependent hypercortisolism, Trans-sphenoidal surgery, Canine, Complications, Addison’s disease, Hypoadrenocorticism

## Abstract

**Background:**

Pituitary-dependent hypercortisolism (PDH) is one of the most common endocrine disorders in veterinary medicine. However, there are few reports on pituitary tumor apoplexy (PTA) in dogs and no reports on its surgical intervention in veterinary medicine. Accordingly, the appropriate treatment is unknown. Herein, a case of PDH and PTA in a dog treated surgically is described.

**Case presentation:**

A mongrel female dog (spayed; age, 8 years and 8 months; weight, 6.1 kg) with persistently elevated alkaline phosphatase underwent adrenocorticotropic hormone (ACTH) stimulation testing (post-stimulation cortisol: 20.5 μg/dL), abdominal ultrasonography (adrenal gland thickness: left, 5.7 mm; right, 8.1 mm), and brain magnetic resonance imaging (MRI) (pituitary-to-brain ratio [PBR], 0.61) at the referral hospital, resulting in a diagnosis of PDH (day 0). On day 9, the dog visited XXXX for the preparation of pituitary surgery to treat PDH. However, on days 10–15, the dog developed a loss of energy and appetite, bloody diarrhea, vomiting, and a decreased level of consciousness. However, on day 16, the dog’s condition recovered. A preoperative MRI scan performed on day 52 (the day of surgery) showed apoplexy in the dorsal pituitary region (PBR, 0.68). Based on the PTA findings, the risks of surgery were described to the owner, and approval was obtained. At the time of trans-sphenoidal surgery, a partial pituitary resection was performed with preservation of the PTA area due to adhesions between the PTA area of the right side of the pituitary and surrounding tissues. The resected pituitary tissue was diagnosed as an ACTH-producing adenoma, with necrotic and hemorrhagic findings. As of day 290, endogenous ACTH and cortisol levels did not exceed the reference range.

**Conclusions:**

The acute signs that occurred on days 10–15 were most likely caused by PTA. Therefore, when signs similar to those detected in acute hypoadrenocorticism are observed in dogs with PDH, it is necessary to include PTA as a differential diagnosis. Trans-sphenoidal surgery may be effective in PDH-affected dogs that develop PTA, but careful attention should be paid to tissue adhesions secondary to hemorrhage that may occur after PTA.

## Background

Pituitary adenomas are the most common cause of pituitary apoplexy in humans. Pituitary tumor apoplexy (PTA) occurs in 0.6–7% of pituitary adenoma cases, but many cases are undiagnosed and thus overlooked [1, 2]. Common clinical signs of PTA in humans include headache (84–100%), nausea (80%), diminished visual acuity (56%), reduced temporal visual field (34–70%), some degree of ophthalmoparesis (45–57%), and impaired mental status (13–30%) [2–5]. Additionally, infarction or necrosis of the pituitary mass usually leads to permanent hypopituitarism in 70–80% of patients with PTA [2, 3, 6]. While the pathophysiology of PTA is not fully understood, it is believed that an enlarged pituitary mass may compress hypophyseal vessels or cause insufficient blood supply, resulting in infarction or hemorrhage within the tumor [7, 8]. Risk factors for PTA include macroadenoma, a pituitary mass invading the cavernous sinus, extreme hypertension or hypotension, dynamic endocrine testing (DET) including insulin tolerance, corticotrophin-releasing hormone (CRH), gonadotropin-releasing hormone, and thyroid-stimulating hormone tests, cardiac surgery, and anticoagulation therapy or anticoagulant status [1, 2]. PTA, which is typically diagnosed using magnetic resonance imaging (MRI) [8], is often treated by surgical decompression and medical management with fluid and hormone replacement therapy [1, 2, 9]. Among clinically stable patients, conservative treatment is considered the appropriate approach. Surgical decompression is mainly directed at the mass effect in the acute phase and can prevent the progression of vision loss, other serious neurological abnormalities, and partial or complete hypopituitarism [1, 2, 9].

Recently, a number of cases of PTA due to pituitary-dependent hypercortisolism (PDH) caused by pituitary macroadenomas have been reported in dogs [10–12]. In eight of these cases, the dogs were euthanized due to severe seizures or death during computed tomography imaging [10–12]. In one case, trilostane medication was discontinued after the onset of PTA, and there was no recurrence of clinical signs during the 6-month follow-up period. Another study, including 19 dogs with suspected PTA, reported a median survival time of 7 months (range: 0–102 months) from the time of PTA diagnosis [12]. However, to date, no reports on pituitary surgery in dogs after suspected PTA exist.

Here, we describe our experience with surgical intervention for PDH with PTA in a dog, and the follow-up findings are presented.

## Case presentation

**Fig. 1:**
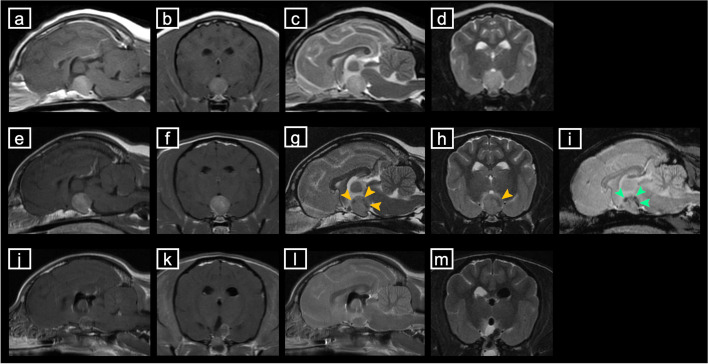
Median sagittal and axial MRI sections on days 0 (before TSS) and 52 (after TSS). **a** MRI-T1 gadolinium angiography on day 0, median sagittal section. The pituitary gland is observed as extending beyond the dorsum sellae and up to the third ventricle, touching the optic chiasm and/or mammillary body but without touching the interthalamic adhesion. The MRI-based classification was considered to be grade III [15]. **b** MRI-T1 gadolinium angiography on day 0, median axial section. The pituitary-to-brain ratio was 0.61 [14], and the pituitary volume was 476 mm^3^, indicating enlargement of the pituitary gland. **c** T2-weighted MRI on day 0, median sagittal section. **d** T2-weighted MRI on day 0, median axial section. **e** Preoperative MRI-T1 gadolinium angiography on day 52, median sagittal section. The MRI-based classification was considered to be grade III. **f** Preoperative MRI-T1 gadolinium angiography on day 52, median axial section. The pituitary-to-brain ratio was 0.68 [14], indicating enlargement of the pituitary gland. The pituitary volume was 476 mm^3^. **g** Preoperative T2-weighted MRI on day 52, median sagittal section. Yellow arrowheads indicate multiple low-signal areas within the pituitary gland. **h** Preoperative T2-weighted MRI on day 52, median axial section. The yellow arrowhead indicates a low-signal area within the pituitary gland. **i** Preoperative T2*-weighted MRI on day 52, median axial section. Green arrowheads indicate multiple low-signal areas within the pituitary gland. **j** Postoperative MRI-T1 gadolinium angiography on day 52, median sagittal section. **k** Postoperative MRI-T1 gadolinium angiography on day 52, median axial section. The pituitary-to-brain ratio was 0.42 [14], and the pituitary volume was 168 mm^3^. **l** Postoperative T2-weighted MRI on day 52, median sagittal section. **m** Postoperative T2-weighted MRI on day 52, median axial section. MRI: magnetic resonance imaging, TSS: trans-sphenoidal surgery

The dog was a spayed female mongrel (age, 8 years and 8 months; weight, 6.1 kg). Due to persistently elevated alkaline phosphatase (ALP) and polyuria polydipsia, which lasted 1 year and 8 months, the dog underwent the following tests at the referral hospital: 1) adrenocorticotropic hormone (ACTH) stimulation testing (pre-stimulation cortisol, 1.2 μg/dL [reference range: 1.0–7.7 μg/dL]; post-stimulation cortisol, 20.5 μg/dL [reference range: 1.0–18.0 μg/dL]), 2) abdominal ultrasonography (left adrenal gland thickness, 5.7 mm; right adrenal gland thickness, 8.1 mm [reference range: 3.7 ± 0.6 mm] [13]), and 3) brain MRI (pituitary-to-brain ratio [PBR], 0.61 [reference: > 0.31]) [14]. Accordingly, the diagnosis of PDH was confirmed (day 0). The initial brain MRI (day 0) was performed using a 0.4-T superconducting MR imaging system (APERTO Eterna; FUJIFILM Healthcare Systems Corporation, Tokyo, Japan) with a slice thickness of 3 mm, a slice gap of 0.5 mm, and a field-of-view of 16 cm. The MRI-based classification was grade III [15], and there were no obvious signs of PTA at this time (Fig. 1).

On day 9, the dog visited Department of Pituitary Surgery, Veterinary Medical Teaching Hospital, Nippon Veterinary and Life Science University for the preparation of pituitary surgery to treat PDH. The dog’s complete blood count (CBC) on day 9 showed no obvious stress steroid pattern or abnormal values (Table 1). In contrast, the biochemical blood test showed elevated levels of ALP (4278 U/L), lipase (221 U/L), and triglycerides (229 mg/dL) (Table 2). Blood endocrinology testing revealed endogenous ACTH (30.1 pg/dL) levels within the reference range, but high ACTH (564 pg/dL) levels post-stimulation with CRH (Table 3) [16]. On day 10, the dog showed a sudden loss of energy and appetite, frequent bloody diarrhea and vomiting, and a decreased level of consciousness. As no improvement was observed on day 11, the dog was referred back to the referral hospital and treated with maropitant (Selenia Injection, Zoetis Inc., 1 mg/kg, sc) for gastrointestinal signs. The dog was also treated with bismuth subnitrate (Diabuster, Kyoritsu Seiyaku Corporation, 200 mg/head, po, bid) and cimetidine (Sawai Pharmaceutical Co., Ltd., 8.3 mg/kg, po, bid) on days 12–15.

**Table 1:** Results of the complete blood count performed at Department of Pituitary Surgery, Veterinary Medical Teaching Hospital, Nippon Veterinary and Life Science University

	Unit	Day 9 (First visit)	Day 16	Day 51 (Pre-TSS)	Day 58 (Post-TSS 6 days)	Day 64 (Post-TSS 12 days)	Day 104 (Post-TSS 52 days)	Day 240 (Post-TSS 188 days)	Reference range
RBC	× 10^6^/μl	7.45	8.37	7.39	6.11	5.81	6.58	6.26	5.5–8.5
PCV	%	52.3	58.2	51.3	47	42.2	47.5	45.9	37–55
Hb	g/dl	17.3	19.3	17.6	14.8	14.7	15.7	15.4	12–18
MCV	fl	70.2	69.5	69.4	71.0	72.6	72.2	73.3	60–77
MCHC	g/dl	33.1	33.2	34.3	34.1	34.8	33.1	33.6	32–36
WBC	/μl	9700	10,700	8900	11,100	14,600	14,600	11,000	6000–17,000
Band	/μl	0	0	0	–	–	0	0	0–300
Seg	/μl	7663	9016	7788	–	–	9928	9790	3000–11,500
Lym	/μl	1358	893	534	–	–	1606	660	1000–4800
Mon	/μl	485	362	267	–	–	2190	495	150–1350
Eos	/μl	97	429	312	–	–	876	55	100–1250
Bas	/μl	97	0	0	–	–	0	0	rare
PLT	×10^4^/μl	23.4	27.6	34.9	33.3	19.6^a^	51.4	41.0	20–50

*TSS* Trans-sphenoidal surgery, *RBC* Red blood cell, *PCV* Packed cell volume, *Hb* Hemoglobin, *MCV* Mean corpuscular volume, *MCHC* Mean corpuscular hemoglobin concentration, *WBC* White blood cell, *Band* Band neutrophil, *Seg* Segmented neutrophil, *Lym* Lymphocyte, *Mon* Monocyte, *Eos* Acidophilic leukocyte, *Bas* Basophilic cell, *PLT* Platelets

^a^ We confirmed the distribution of platelets as aggregated and the presence of sufficient numbers of platelets in the blood on the glass slide

**Table 2:** Results of the biochemical blood test performed at Department of Pituitary Surgery, Veterinary Medical Teaching Hospital, Nippon Veterinary and Life Science University

	Unit	Day 9 (First visit)	Day 16	Day 51 (Pre-TSS)	Day 58 (Post-TSS 6 days)	Day 64 (Post-TSS 12 days)	Day 240 (Post-TSS 188 days)	Reference range
LDH	U/L	63	68	104	58	85	121	20–119
AST	U/L	17	18	21	12	38	27	14–44
ALT	U/L	31	32	40	77	111	82	14–68
ALP	U/L	4278	1633	3307	3261	3785	7499	47–254
GGT	U/L	5	6	5	23	17	19	2–15
T-BIL	mg/dL	0.1	0.1	0.1	0.1	0.1	0.1	0–0.2
D-BIL	mg/dL	0	0	0	0	0	0.1	0–0.1
TBA	μmol/L	0.8	0.9	9.6	4.3	48.0	7.2	0.1–20.0
TP	g/dL	6.9	5.9	6.8	6.4	5.8	6.7	4.9–7.2
ALB	g/dL	3.5	2.9	3.6	3.3	3.1	3.5	2.0–3.2
CK	U/L	72	72	73	38	91	102	47–168
AMY	U/L	700	722	717	476	534	664	248–2284
LIP	U/L	221	386	233	401	488	599	16–160
BUN	mg/dL	14.4	14.7	18.9	48.7	34.2	15.7	9.2–29.2
CRE	mg/dL	0.67	0.74	0.68	0.94	0.49	0.66	0.40–1.45
Ca	mg/dL	10.4	10.2	10.7	11.1	10.0	10.9	9.1–12.3
IP	mg/dL	2.7	2.7	3.3	6.5	4.5	3.2	1.9–5.0
GLU	mg/dL	111	154	99	83	102	115	75–128
TG	mg/dL	229	129	303	337	1272	164	17–113
T-CHO	mg/dL	261	165	258	219	235	254	105–322
Na	mEq/L	146	142	146	147	144	143	141–152
K	mEq/L	4.1	4.2	4.8	4.2	3.8	4.2	3.8–5.1
Cl	mEq/L	109	109	108	99	103	98	102–117
CRP	mg/dL	0.18	0.97	0.15	0.81	0.27	0.13	0–1.00

*TSS* Trans sphenoidal surgery, *LDH* Lactate dehydrogenase, *AST* Aspartate aminotransferase, *ALT* Alanine aminotransferase, *ALP* Alkaline phosphatase, *GGT* γ- glutamyltransferase, *T-BIL* Total bilirubin, *D-BIL* Direct bilirubin, *TBA* Total bile acid, *TP* Total protein, *ALB* Albumin, *CK* Creatine kinase, *AMY* Amylase, *LIP* Lipase, *BUN* Blood urea nitrogen, *CRE* Creatinine, *Ca* Calcium, *IP* Inorganic phosphorus, *GLU* Glucose, *TG* Triglyceride, *T-CHO* Total cholesterol, *CRP* C-reactive protein

**Table 3:** Results of the blood endocrinology results performed at Department of Pituitary Surgery, Veterinary Medical Teaching Hospital, Nippon Veterinary and Life Science University

	Unit	Day 9 (First visit)	Day 51 (Pre-TSS)	Day 58 (Post-TSS 6 days)	Day 64 (Post-TSS 12 days)	Day 104 (Post-TSS 52 days)	Day 240 (Post-TSS 188 days)	Reference range
CRH test								
Endogenous ACTH	pg/dL	30.1	28.5	13.1	12.8	< 0.1	7.4	5.0–36.0
Post-stimulation ACTH	pg/dL	564.0	–	–	–	–	–	1.9–153.4^a^
ACTH stimulation test								
Endogenous cortisol		1.23	–	0.78	0.10	< 0.1	< 0.1	1.0–7.8
Post-stimulation cortisol	μg/dL	–	–	–	7.65	–	–	5.0–20.0
T4	μg/dL	1.95	–	< 0.50	0.55	4.37	1.21	1.1–3.6
FT4	ng/dL	1.23	–	< 0.30	0.43	4.02	0.77	0.50–3.00
TSH	ng/dL	0.20	–	0.04	–	< 0.03	0.06	0.08–0.32

*TSS* Trans-sphenoidal surgery, *CRH* Corticotropin-releasing hormone, *ACTH* Adrenocorticotropic hormone, *T4* Thyroid hormone, *FT4* Free thyroxine hormone, *TSH* Thyroid-stimulating hormone

^a^ Normal dog data obtained from Tanaka et al. [14]

On day 16, the dog recovered and came back to our facility. The CBC test on day 16 revealed lymphopenia (Table 1), and the blood and biochemical tests showed similar findings to those on day 9 (Table 2). Physical examination found that the capillary refill time was < 1 sec, turgor response was < 1 sec, and both visible mucosa coloration and femoral artery pressure were normal. At this time, no endocrinological or neurological examinations were performed because the occurrence of PTA was not included in the list of differential diagnoses. Therefore, only general blood and physical examinations were performed to minimize stress, and the dog was returned to the owner. At the owner’s request and given the clinical improvement, brain MRI and trans-sphenoidal surgery (TSS), which had been scheduled to be performed on day 22, were postponed to day 52. The dog had no gastrointestinal signs from days 16–51 and was hospitalized on day 51 in preparation for pituitary surgery on day 52.

The CBC test on day 51 revealed lymphopenia (Table 1), and the results from the biochemical blood tests were similar to those on days 9 and 16 (Table 2). However, on day 51, the endogenous ACTH level was high (225.0 pg/dL) (Table 3). On day 52, preoperative brain MRI showed a PBR of 0.68 [14], which was slightly higher than that on day 0. Brain MRI on day 52 was performed using a 3.0-T superconducting MR imaging system (Signa HDxt; GE Healthcare, Tokyo, Japan) with a slice thickness of 2 mm, no slice gap, and a field-of-view of 15 cm. The MRI-based classification was grade III [15], which was the same as observed on day 0. The pituitary volume was 448 mm^3^ (slice thickness, 2 mm), and there were multiple low-signal regions within the pituitary on mid-sagittal and axial sections of the T2-weighted image. Additional T2*-weighted imaging revealed a low-signal region in the right dorsal part of the pituitary mass, suggesting the presence of hemorrhage, and the hemorrhagic area was adjacent to the arterial cerebral circle of Willis (Fig. 1). Based on these findings, we explained the risks of surgery to the owner again, and after obtaining their approval, TSS was performed on that day (day 52).

TSS was performed in accordance with the method reported by Meij and colleagues [17, 18]. Briefly, a burr hole was made in the basisphenoid bone, and the ventral aspect of the pituitary gland was exposed. Next, a dural incision was performed, and the pituitary gland was resected using fine neurosurgical grasping forceps. For areas that could not be easily removed, suction resection was attempted using a 2 mm suction cannula. At this time, the right side of the pituitary mass, which was dark red, could not be resected by suction as it was adherent to the surface of the third ventricle. Adhesion to the surrounding tissues due to PTA was suspected, and the adherent tissue was adjacent to the arterial cerebral circle of Willis. To avoid catastrophic damage to the arterial vasculature, suction resection was completed as a partial resection.

**Fig. 2:**
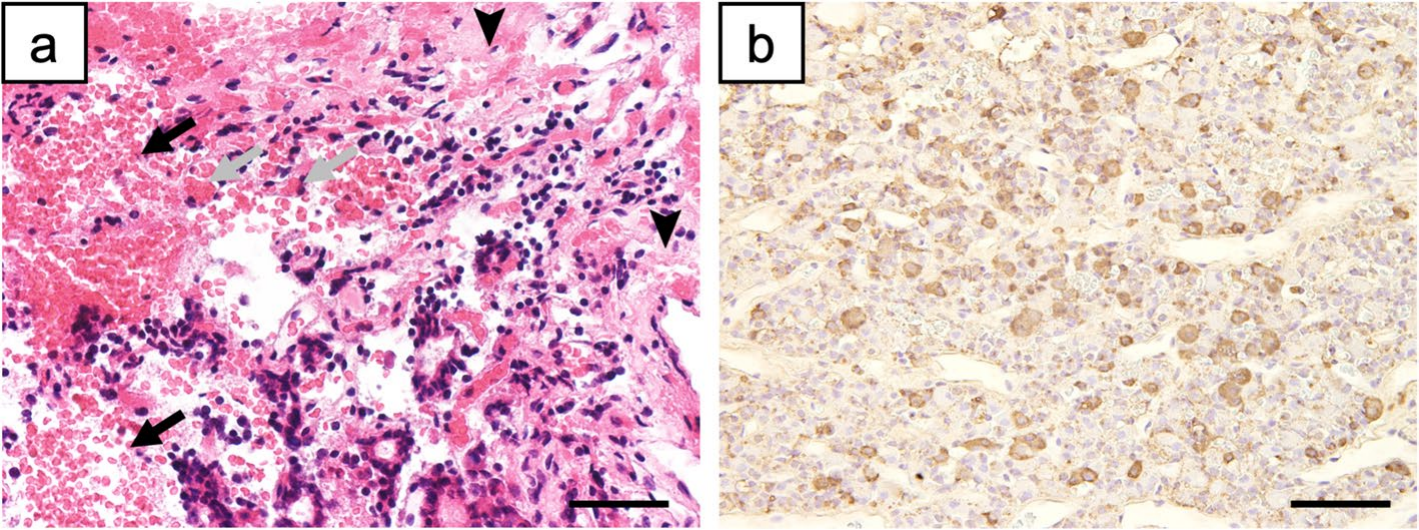
Histopathological examination of the pituitary mass removed on day 52. **a** Hematoxylin & eosin-stained image. The arrowheads indicate necrotic findings in the glandular pituitary region. The black arrows indicate hemorrhage, with accumulation of erythrocytes outside the blood vessels. The grey arrows indicate macrophages, with phagocytosis of red blood cells. **b** Anti-ACTH-immunostained image. The reddish-brown area is ACTH-positive, indicating that the resected pituitary tissue is an ACTH-producing adenoma. Scale bars: 20 μm. ACTH: adrenocorticotropic hormone

Immediate postoperative brain MRI showed that the remaining pituitary volume was 168 mm^3^, with a resection rate of 62.5% (Fig. 1). Histopathological examination of the resected pituitary mass found necrosis of the glandular pituitary region and hemorrhage, with accumulation of erythrocytes outside the vessels. Further analysis using hematoxylin & eosin staining demonstrated the presence of macrophages with phagocytosis of erythrocytes. In addition, immunostaining revealed numerous areas positive for ACTH (Fig. 2). Based on these immunohistopathological findings, the diagnosis of ACTH-producing pituitary adenoma with hemorrhage and necrosis associated with PTA was confirmed.

Hormone replacement therapy was administered from the postoperative period to day 290 (238 days postoperatively). This treatment included desmopressin acetate for antidiuretic hormone replacement [17, 19, 20], hydrocortisone and prednisolone for adrenocortical hormone replacement [17, 19, 20], levothyroxine sodium for thyroid hormone replacement [17, 19, 20], and ethinylestradiol for estrogenic hormone replacement [21] (Table 4). Ethinylestradiol was administered due to urinary incontinence that developed after discharge and was believed to be due to estrogen deficiency; the urinary incontinence disappeared the day after the medication was started. The dog’s postoperative consciousness and physical condition were stable, and the dog was discharged from our facility on day 67 (15 days postoperatively). During the postoperative period, central diabetes insipidus caused by the TSS persisted, requiring continuous antidiuretic hormone medication. According to the findings from postoperative CBC (Table 1) and biochemical blood (Table 2) tests on day 240, the dog had lymphopenia, and the ALP and lipase levels were twice as high as before surgery. In contrast, endogenous ACTH and cortisol levels were not elevated beyond the reference range (Table 3). In addition, the dog received oral prednisolone twice daily without dosage change as a glucocorticoid replacement, and this treatment was ongoing when this manuscript was prepared (Table 4).

**Table 4:** Hormone replacement therapy in the postoperative period

Function	Medicine	Manufacturer	Period	Dose / administration method
Antidiuretic hormone	Desmopressin acetate	Kissei Pharmaceutical CO., LTD	Immediately after TSS to 5 days after TSS	0.1 μg/kg, sc, bid
	Desmopressin acetate	Kissei Pharmaceutical CO., LTD	6 days after TSS to present	0.1 μg/kg, os, bid
Adrenocortical hormone	Hydrocortisone	Nichi-Iko Pharmaceutical CO., LTD	Immediately after TSS to 1 day after TSS	1.0 mg/kg, iv, qid
	Prednisolone	Yoshindo Co., Ltd.	2 days after TSS to present	0.1–0.2 mg/kg, po, bid
Thyroid hormone	Levothyroxine sodium	ASKA Pharmaceutical CO., LTD.	7 days before TSS to present	20–30 μg/kg, po, bid
Estrogenic hormone ·	Ethinylestradiol	ASKA Pharmaceutical CO., LTD.	62 days after TSS to present	0.03 mg/kg, po, sid

*TSS* Trans-sphenoidal surgery

## Discussion and conclusions

In recent years, cases of pituitary macroadenomas secondary to PTA have been reported in veterinary medicine; however, no effective treatment has been identified, and no case of surgical intervention with long-term prognostic follow-up has been reported [8, 10–12]. In the present case, T2*-weighted MRI on day 52 showed that the hemorrhage was concentrated in the dorsal portion of the pituitary tumor [22], and the ACTH concentration measured on day 51 was similar to that on day 9, suggesting that the ventral pituitary tissue maintained ACTH production. Furthermore, since the pituitary gland was clearly enlarged, TSS was performed to reduce the mass effect [22]. If the pituitary mass had been left untreated, there would have been a risk of further growth of the adenoma and consequently a further increase in the size of the mass, possibly inducing impairment of thalamic function. Furthermore, if the mass had occupied the third ventricle, obstructive hydrocephalus might have followed, resulting in acute intracranial hypertension and subsequent brain herniation. TSS was selected to prevent this catastrophic development. In the present case of PTA in a dog, surgical treatment with total removal of the pituitary mass was planned, but partial pituitary resection was performed because of adhesions with the surrounding tissue due to PTA and because the adherent tissue was adjacent to the arterial cerebral circle of Willis. The forcible removal of the adherent tissue would have risked the rupture of the circle of Willis, which may lead to catastrophic consequences, even intra- or postoperative death. There is insufficient evidence to support that TSS contributed to the clinical improvement in this dog; however, the pituitary mass volume was effectively reduced. In human medicine, TSS for PTA is directed at the mass in the acute phase with the progression of severe clinical signs, such as visual disturbances or headaches [1, 2, 9]. However, in the present case, TSS was performed approximately 40 days after the clinical signs of PTA were suspected (on day 10), which might have provided the time for the adhesions to occur because of chronic inflammation due to clots and necrotic tissues. Therefore, when removing a pituitary mass in dogs after the onset of PTA, the time elapsed after PTA onset should be taken into consideration, and sufficient attention should be paid to tissue adhesions secondary to hemorrhage, especially around the arterial blood vessels (internal carotid artery, caudal communicating artery, and arterial cerebral circle of Willis).

The acute clinical signs observed on the 10th day of illness were unlikely to be due to medically induced Addison’s disease, as the dog was not medicated with trilostane. As such, clinical signs of pancreatitis or enteritis were considered rather than other possible diagnoses due to stress at the time of the visit and from the examination. Accordingly, blood endocrinological tests and neurological examinations were not performed when the dog returned to our facility on day 16, and brain MRI and TSS were postponed. In the present case, our lack of knowledge of and experience with PTA may have led to a delay in therapeutic interventions including surgery. Therefore, in veterinary medicine, especially when clinical signs of hypopituitarism are observed in PDH cases, it is necessary to include PTA as one of the differential diagnoses. Furthermore, a rapid diagnosis is essential for the most effective PTA treatment in the acute phase [7, 23]. In humans, brain MRI is the best diagnostic method because it can confirm pituitary hemorrhage in 88–91% of cases [8, 24], and it is also reported to be effective in the early detection of PTA [25]. In veterinary medicine, it is also important to quickly diagnose acute PTA to provide the most appropriate treatment, and brain MRI may be an effective tool for diagnosing acute cases of suspected PTA. Notably, the MRI performed at the referral hospital on day 0 was conducted using a 0.4-T scanner, while the MRI at our facility on day 52 was conducted using a 3.0-T scanner. Given the difference in magnetic field strength, some details might have been missed in the initial MRI, suggesting that an MRI scanner with a higher magnetic field strength should be used in future cases.

In humans, 70–80% of patients with PTA develop permanent or temporary hypopituitarism, and approximately three-quarters of patients recovering from PTA require anterior pituitary hormone replacement therapy [2, 3, 6]. In addition, glucocorticoids, thyroid hormones, and desmopressin are required long-term in 60–80, 60%, and 10–15% of patients with PTA, respectively [4]. In the present case, the endogenous ACTH and cortisol levels had never risen above the reference range, even though the pituitary tissue in the PTA area was preserved during TSS. Therefore, based on MRI and surgical findings, the preserved pituitary tissue in this case might have developed PTA; although a small amount of secreted ACTH remained, it was not enough to cause HGC. One possibility is that the lack of increased ACTH production in the postoperative period beyond the reference range, despite the presence of residual PTA tissue, may be caused by the loss of residual blood supply to the PTA area due to partial resection of the pituitary. The possibility that the unmeasurably low plasma ACTH and serum cortisol levels on day 104 can be explained by iatrogenic secondary hypoadrenocorticism due to daily prednisolone administration cannot be ruled out.

Although DET, including the use of CRH preparations, is a risk factor for PTA in humans [1, 2], it remains unclear whether it is also a risk factor in dogs. In the present case, a CRH test was performed at the time of the first visit to our facility (day 9). In addition, on day 10, the dog had clinical signs that were very similar to those reported as common PTA symptoms in humans [2–5], and on day 52, a brain MRI revealed a hemorrhagic lesion within the pituitary mass. Taken together, these findings suggest that DET, such as the CRH test, may be a risk factor for PTA in dogs. Thus, DET in dogs with PDH and suspected macroadenoma should be performed with extreme caution.

In this study, both an ACTH stimulation test and a CRH test were performed for the diagnosis of PDH. The ACTH stimulation test is the most common endocrine screening test for hyperadrenocorticism (HAC) in veterinary medicine [26–28], and it is considered superior to the low-dose dexamethasone suppression test in terms of simplicity and specificity [29, 30]. A recent report also suggests that the CRH test, which can assess both endogenous and post-stimulation ACTH concentrations, may be effective in differentiating PDH from cortisol-producing adrenal tumor [16]. However, a low-dose dexamethasone suppression test could have been used in this case to determine HAC with higher sensitivity [29, 30].

The present case study has several limitations. First, it was impossible to perform blood endocrinological tests near the time of the clinical signs of the suspected PTA. Therefore, the endocrinological status at that time could not be accurately assessed. The only blood parameter supporting that the reduced serum cortisol levels were caused by the suspected PTA is the sudden drop in serum ALP observed on day 16. Second, it was impossible to perform neurological examinations at the time of the clinical signs of suspected PTA. Therefore, the degree of neurological damage could not be accurately assessed. Third, all postoperative hormone tests were performed under continued glucocorticoid replacement, and the ACTH stimulation test was only performed at one postoperative time point. Therefore, it cannot be ruled out that hormone replacement therapy, in this case, is being administered in addition to remnant continued endogenous secretion of pituitary hormones.

In conclusion, TSS is considered safe in dogs with PDH complicated by PTA when careful attention is paid to tissue adhesions secondary to hemorrhage that may develop after PTA. However, it remains to be investigated when, in relation to PTA, it is appropriate to perform TSS.

## Data Availability

The data supporting the conclusions of this article are included within the article.

## Abbreviations

ACTH: Adrenocorticotropic hormone
ALP: Alkaline phosphatase
CBC: Complete blood count
CRH: Corticotrophin-releasing hormone
DET: Dynamic endocrine testing
MRI: Magnetic resonance imaging
PBR: Pituitary-to-brain ratio
PDH: Pituitary-dependent hyperadrenocorticism
PTA: Pituitary tumor apoplexy
TSS: Trans-sphenoidal surgery
